# High-effective approach from amino acid esters to chiral amino alcohols over Cu/ZnO/Al_2_O_3_ catalyst and its catalytic reaction mechanism

**DOI:** 10.1038/srep33196

**Published:** 2016-09-13

**Authors:** Shuangshuang Zhang, Jun Yu, Huiying Li, Dongsen Mao, Guanzhong Lu

**Affiliations:** 1 Research Institute of Applied Catalysis, School of Chemical and Environmental Engineering, Shanghai Institute of Technology, Shanghai 201418, China; 2 Key Laboratory for Advanced Materials and Research Institute of Industrial catalysis, East China University of Science and Technology, Shanghai 200237, China

## Abstract

Developing the high-efficient and green synthetic method for chiral amino alcohols is an intriguing target. We have developed the Mg^2+^-doped Cu/ZnO/Al_2_O_3_ catalyst for hydrogenation of L-phenylalanine methyl ester to chiral L-phenylalaninol without racemization. The effect of different L-phenylalanine esters on this title reaction was studied, verifying that Cu/ZnO/Al_2_O_3_ is an excellent catalyst for the hydrogenation of amino acid esters to chiral amino alcohols. DFT calculation was used to study the adsorption of substrate on the catalyst, and showed that the substrate adsorbs on the surface active sites mainly by amino group (-NH_2_) absorbed on Al_2_O_3_, and carbonyl (C=O) and alkoxy (RO-) group oxygen absorbed on the boundary of Cu and Al_2_O_3_. This catalytic hydrogenation undergoes the formation of a hemiacetal intermediate and the cleavage of the C–O bond (rate-determining step) by reacting with dissociated H to obtain amino aldehyde and methanol ad-species. The former is further hydrogenated to amino alcohols, and the latter desorbs from the catalyst surface.

Chiral amino alcohols, especially L-phenylalaninol, are very important one of chiral organic compounds, and have been widely applied as the building blocks in biologically active molecules, pharmaceutical synthesis, fine chemicals as well as resolution of racemic mixtures, *etc*.^1^,^2^,^3^,^4^. Traditionally, amino alcohols were synthesized by homogeneous hydrogenation of amino acids with metal hydrides^5^,^6^ or the catalytic aminolysis of epoxides with amines^7^,^8^. But these methods above or processes suffer from production of a large amount of salts, using large excessive reagents, and high production cost, which make them less attractive for industrial and technical applications.

Undoubtedly, the heterogeneous catalytic hydrogenation with H_2_ as a reducing reagent is the most interesting catalytic method for synthetic organic chemistry, which is an environmentally benign, easier separation and re-used process for the catalysts. For instance, the Nishimura catalyst consisted of Rh/Pt oxide could be used in the hydrogenation of chiral α-hydroxy or α-amino esters to the corresponding diols or amino aclohols without racemization^9^. However, the application of this catalyst is limited, because that the phenyl group in the reaction substrates can be also catalytic hydrogenated simultaneously. Ru/C can be used as the catalyst for the aqueous-phase catalytic hydrogenation of amino acids to amino alcohols, but the drawbacks of its low activity and loss of optical purity could not be overcome^10^,^11^. Tamura *et al*. reported that Rh-MoO_x_/SiO_2_ was an effective catalyst for direct hydrogenation of various amino acids to amino alcohols with high yields (90–94%) in a water solvent at 313–323 K^12^. However, these noble metal catalytic systems mentioned above are also not commercially available, due to the high loading amount of noble metals and relatively poor reusability of the Rh-based catalyst.

Recently, we have developed one kind of novel Cu/ZnO/Al_2_O_3_ catalyst for synthesis of chiral L-phenylalaninol by the catalytic hydrogenation of L-phenylalanine methyl ester at mild reaction condition^13^,^14^,^15^. Comparing with the traditional Cu-ZnO-Al_2_O_3_ hydrogenation catalysts^16^,^17^, the composition, active phase structure and preparation method of our catalyst have been completely modified. This Cu/ZnO/Al_2_O_3_ catalyst was further optimized by doping Mg, and the high yield of L-phenylalaninol (91.2%) without racemization was achieved^15^. For this highly efficient and stabilized CuZn_0.3_Mg_0.1_AlO_x_ catalyst for hydrogenation of L-phenylalanine methyl ester to chiral L-phenylalaninol, whether it can be used as the catalyst for the hydrogenation of other amino acid esters? What kind of substrate structure is easily hydrogenated? What is this catalytic reaction mechanism for hydrogenation of amino acid esters over this Cu/ZnO/Al_2_O_3_ catalyst? It is very necessary to solve these questions above, no matter in the academic research or commercial utilization. Clarification of the catalytic reaction mechanism is helpful to improve the design of the new catalyst from the hydrogenation of amino acid ester or similar reaction substrates to form the chiral L-phenylalaninol or similar chiral compounds. For the identical hydrogenation with different substrates, because of their different electronic structure, adsorption mode and geometrical restrictions, the identical catalyst also exhibits different catalytic performance. In different chiral amino acid esters, there are different functional groups, such as the amino group, ester alkoxy group, *etc*., and these functional groups would affect obviously the catalytic performance of the catalyst^9^,^18^,^19^,^20^. At the same time, whether these functional groups in chiral amino acid esters affect the catalytic reaction mechanism need also to be clarified.

Herein, a series of L-phenylalanine esters with different ester group substituents (or protection of amino group) were used as the reaction substrates, to study the adaptability or versatility of the CuZn_0.3_Mg_0.1_AlO_x_ catalyst for their hydrogenation to chiral L-phenylalaninol. Based on the results above, the reactivities of various reaction substrates over this catalyst would be compared. Furthermore, density functional theory (DFT) calculation, served as a powerful tool for studying the catalysis and reaction mechanism^21^, was used to investigate the adsorption configuration of the substrate molecules with different groups on the catalyst surface and explore the possible reaction pathways of synthesizing L-phenylalaninol by L-phenylalanine methyl ester hydrogenation, to further validate our experiment results. Combined with experimental and theoretical study, the catalytic reaction mechanism over the CuZn_0.3_Mg_0.1_AlO_x_ catalyst for the title hydrogenation was clearly demonstrated.

## Results and Discussion

### Effect of the substrate on its catalytic hydrogenation

The effect of the amino group of substrate on the catalytic hydrogenation over the CuZn_0.3_Mg_0.1_AlO_x_ catalyst is compared in Table 1. It can be seen that 90.1% conversion and 95.1% selectivity of L-phenylalaninol were obtained when L-phenylalanine methyl ester (L-p-me) was used as the reaction substrate (entry 1.1). If the amino group of L-p-me protected by the tert-butyloxycarbonyl (t-Boc) group, the conversion and selectivity to L-phenylalaninol were sharply dropped and only 26.3% and 9.5% respectively (entry 1.2). For the substrate without amino group substituent at α-carbon, the catalytic hydrogenation hardly occurred, only 1.3% yield of 3-phenyl propyl alcohol was produced (entry 1.3).

The results in Table 1 indicate that the amino group at the α-position of carboxy group in α-amino acid esters is essential for this catalytic hydrogenation, that is to say, the amino group is probably firstly adsorbed on the active sites of the catalyst surface, then inducing the next step reaction. More specifically, NH_2_ group probably interacts with the oxygen sites on the catalyst surface by hydrogen bonding, or coordinates with metal active sites by the lone electron pair of the nitrogen atom in NH_2_ group, because the α-NH_2_ group can interact with a basic (oxidic) site situated very close to the metallic centers^9^. When the amino group was protected by t-Boc, an adsorption of amino group would be hindered, leading to the drop of reactivity and very low selectivity to the desired product (9.5%), and its by-product was mainly N-Boc-L-phenylalaninol. In addition, only 2.5% conversion was achieved when the amino group was removed from the L-p-me, which suggests that the reaction undergoes towards the other reaction pathway without the adsorption of amino group, resulting in the low reactivity and low selectivity (52%) to the desired product. And the by-product was mainly 3-phenylpropyl aldehyde.

Note when α-amino acid ester was used as the reaction substrate, the highest conversion and 100% *ee* value could be achieved at lower temperature (110 °C), because high temperature is not appropriate for the stabilization of optically active substances and results in a racemization^22^,^23^,^24^. And the hydrogenation of 3-phenylpropionic acid methyl ester was hard to occur at the same temperature due to the absence of NH_2_ group, resulting in very low conversion (2.5%). It can be inferred that the presence of amino group is beneficial to the adsorption of α-amino acid esters at low temperature, which not only improves the catalytic reaction, but also inhibits the racemization of product.

To identify the preferred adsorption sites on the catalyst for the functional groups of substrates, the DFT theoretical calculation for L-p-me adsorption on the Cu_6_/γ-Al_2_O_3_(100) model was performed. With regard to using Cu_6_/γ-Al_2_O_3_(100) instead of real catalysts as the model catalyst, the detailed demonstration can be seen in the computational details of DFT study in the experimental section.

**Figure 1:**
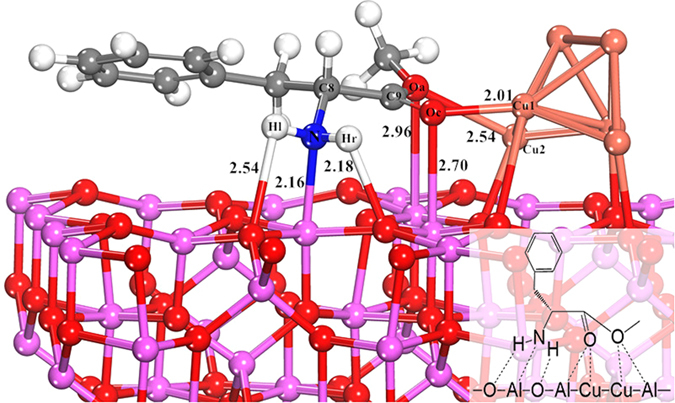
Optimized configuration of L-p-me adsorbed on Cu_6_/γ-Al_2_O_3_(100). (Cu atoms are orange, O atoms are red, Al atoms are pink, C atoms are gray, H atoms are white, N atoms are dark-blue). Bond distances are reported in Å.

The optimized configuration of L-p-me adsorbed on the Cu_6_/γ-Al_2_O_3_(100) catalyst is shown in Fig. 1. The reactant L-p-me interacts with the catalyst surface mainly through two functional groups: the amino group (-NH_2_) of L-p-me adsorbed on the O-Al-O sites of *γ*-Al_2_O_3_(100) facet. The bond distances of H_l_-O, N-Al, H_r_-O are 2.54, 2.16 and 2.18 Å respectively. And the other is the carbonyl group oxygen (O_c_) and alkoxy group oxygen (O_a_) of L-p-me absorbed on the Al and Cu sites situated in the boundary of Cu cluster and Al_2_O_3_. The bond distances of O_c_-Al, O_c_-Cu_1_, O_a_-Al, O_a_-Cu_2_ are 2.70, 2.01, 2.96 and 2.54 Å, respectively. The O_c_ atom linked to the surface Cu_1_ and the O_a_ atom bonded with the surface Cu_2_ is likely to form a bi-dentate configuration. Simultaneously, the O_c_ and O_a_ atoms can also interact with surface Al sites. That is to say, the adsorption of O_c_ and O_a_ are stabilized at the boundary of metal-support. These studies validated our previous proposal that the active site of Cu atoms is situated in the boundary between Cu cluster and the support^14^.

**Figure 2:**
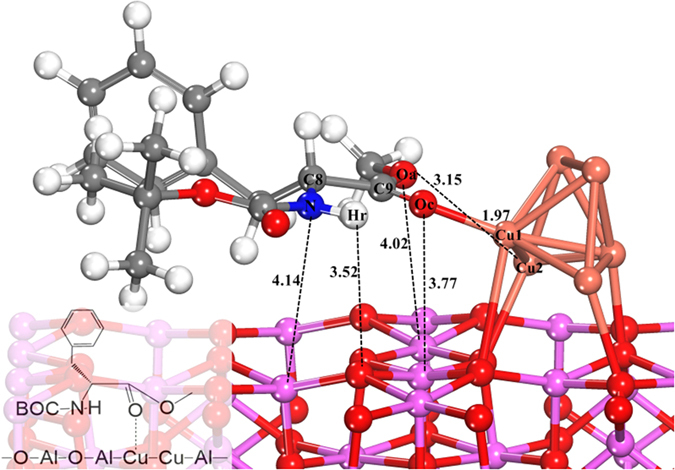
Optimized configuration of N-Boc-L-Phenylalanine methyl ester adsorbed on Cu_6_/γ-Al_2_O_3_(100). The distances between two atoms are reported in Å.

**Figure 3:**
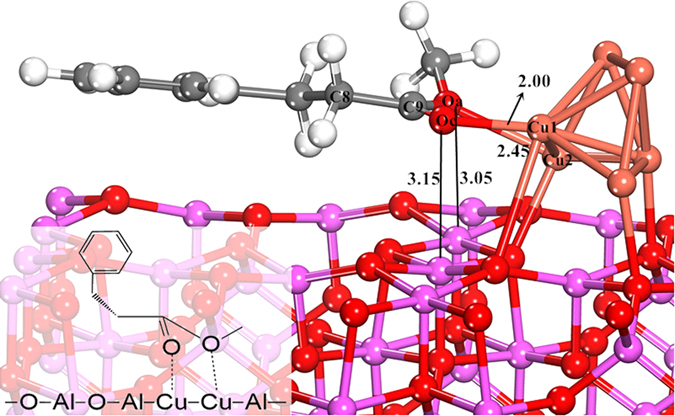
Optimized configuration of 3-phenylpropionic acid methyl ester adsorbed on Cu_6_/γ-Al_2_O_3_(100). The distances between two atoms are reported in Å.

To further explore the role of amino group, the substrates with –NH_2_ protected by a t-Boc group or without the amino group would also be investigated. The DFT optimized configurations of N-Boc-L-phenylalanine methyl ester (L-p-Boc-me) and 3-phenylpropionic acid methyl ester (3-p-a-me) adsorbed on Cu_6_/γ-Al_2_O_3_(100) are shown in Figs 2 and 3. The corresponding structural parameters and adsorption energies are listed in Table 2. The results show that, when the –NH_2_ was protected by t-Boc group, the distance between N and Al atoms is elongated from 2.16 Å to 4.14 Å, and the distance of H_r_-O is increased from 2.18 Å to 3.52 Å, and the corresponding distances of O_a_-Al and O_a_-Cu_2_ are elongated as well. And the adsorption energy of L-p-Boc-me on Cu_6_/γ-Al_2_O_3_ (−0.86 eV) is lower than that of L-p-me on Cu_6_/γ-Al_2_O_3_ (−1.14 eV), which confirms the decrease in its adsorption stability after t-Boc group protection, compared with adsorption state of L-p-me. When the amino group in L-p-me was removed, only O_c_ and O_a_ of 3-p-a-me are bonded with Cu_1_ and Cu_2_ sites respectively, resulting in the most unstable of adsorbed structure because of the lowest adsorption energy of −0.51 eV. Therefore, it is suggested that the amino group binding on Al_2_O_3_ support of Cu_6_/γ-Al_2_O_3_(100) is an important contribution for enhancing the adsorption strength of reactant, further improving the catalytic conversion, which is consistent with the experimental findings.

The effects of the ester group substituents in substrates on the catalytic hydrogenation over the CuZn_0.3_Mg_0.1_AlO_x_ catalyst were tested, and results are listed in Table 3. In the catalytic hydrogenation of L-phenylalanine the yield of L-phenylalaninol can be hardly obtained, and only undesirable side reactions occurred on the base of the substrate conversion (entry 3.1). High yields of the L-phenylalaninol were obtained for hydrogenation of L-phenylalanine methyl ester and L-phenylalanine ethyl ester (entries 3.2 and 3.3). The conversion and product yield were decreased when the substrates have the group with steric hindrance near the ester alkoxy group, such as tert-butyl (t-Bu) and benzyl groups (entry 3.4 and 3.5), which is similar to the hydrogenation of alanine t-Bu ester over Nishimura catalyst^9^ and hydrogenation reduction of benzoic acid esters over the ruthenium complex catalyst^18^. When the L-phenylalanine ester derivatives with the ester groups of trifluoro ethyl or chloro methyl group were used as the reactants, slightly lower conversion of 73.1% and 75.3% were obtained (entries 3.6 and 3.7), in comparison with L-p-me as the reactant.

Combined with the optimized structure of L-p-me adsorbed on Cu_6_/γ-Al_2_O_3_(100) above, we infer that the chemisorption bonds between alkoxy group oxygen atom and active sites on the catalyst surface could be effected by the alkoxy group (RO-). That is to say, the different R groups in the alkoxy group (RO-) can induce the change of electron density of alkoxy group oxygen atom or the steric hindrance in α-amino acids ester, further affecting the catalytic activity or the catalytic reaction. In order to clarify the above supposition, the various reactants or adsorbates were calculated by using DFT, and the corresponding structural parameters and adsorption energies are listed in Table 4. And the optimized configurations of L-phenylalanine esters adsorbed on Cu_6_/γ-Al_2_O_3_(100) are shown in Figs S1, S2, S3, S4 and S5.

As shown in Table 4, for all the structures of L-phenylalanine esters adsorbed on Cu_6_/γ-Al_2_O_3_(100), the bond distance of H_l_-O is 2.50~2.60 Å, the bond distance between N atom and Al atom is 2.16~2.21 Å, and the bond distance of H_r_-O is 2.18~2.33 Å, which has a little difference or almost be the same. It is concluded that the adsorption state between amino group (-NH_2_) and surface O-Al-O sites is hardly effected by the alkoxy groups, because of the relatively far distance between -NH_2_ and -OR.

However, different ester group substituents have a significant influence on the adsorption of alkoxy oxygen in the ester group. The DFT calculations show that the distances of O_a_-Al and O_a_-Cu_2_ increases in the order of the substrates with the substituent of CH_3_ < CH_2_CH_3_ < CH_2_Cl ≈ CH_2_CF_3_, which is consistent with an increase of their electron-withdrawing properties, and the adsorption energy of substrates on the active sites decreases in the same tendency. It was reported that, the higher electron density of aromatic cycle or N-heterocycle caused by the electron-donor of methyl group is favorable for the formation of the hydrogen bond between the surface hydroxyl group and substrate molecule^25^. Thus, we infer that the electron-donating effect of methyl or ethyl increases electron density around the alkoxy group oxygen atom, resulting in an increase in the adsorption energy of the alkoxy group oxygen on the active sites of the catalyst surface. Natal Santiago *et al*. thought that the stronger the ability of R group for donating electron to the alkoxy oxygen atom, the easier the cleavage of the C–O bond in the ester group (O=C–O) is and the lesser the activation energy of its dissociation is^26^. As shown in Table 3, the catalytic reactivity of the substrate increases in the order of its substituent of CH_2_CF_3_ < CH_2_Cl < CH_2_CH_3_ ≈ CH_3_, that is to say, for the most part, the reactivity of the reactant is decreased with a decrease in the electron-donor ability of the R group, which affects the adsorption energy and activation energy for the dissociation of ester group.

On the other hand, the DFT calculations also show that, when the ester group substituent is the group of t-Bu or benzyl, the distance between the oxygen atoms and the active sites on the catalyst surface is elongated and the adsorption energy decreases. It is obviously that the alkoxy group oxygen is difficult to get close to the catalyst surface due to the steric-hindrance effect caused by the bulky group of t-Bu or benzyl, resulting in lower reactivity of L-phenylalanine t-Bu ester and L-phenylalanine benzyl ester. Moreover, no trace of desired product was detected for hydrogenation of L-phenylalanine (entry 3.1). In comparison with the hydrogenations of many carbonyl compounds, the hydrogenation of acids to alcohols is more difficult due to the fact that the carbonyl group has weak polarizability and has, consequently, lower reactivity^27^,^28^,^29^,^30^.

### Study on the reaction mechanism

**Figure 4:**
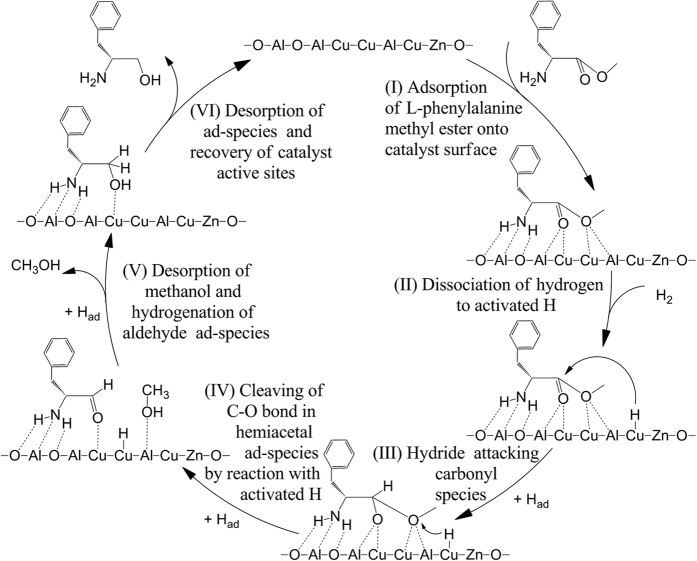
Possible reaction mechanism of the hydrogenation of L-phenylalanine methyl ester over the Cu/ZnO/Al_2_O_3_ catalyst.

Based on the above results and discussions, the reaction mechanism over the Cu/ZnO/Al_2_O_3_ catalyst for the hydrogenation of L-phenylalaine ester, using L-phenylalaine methyl ester as a representative compound, is proposed in Fig. 4.

In the hydrogenation of L-phenylalanine methyl ester over the Cu/ZnO/Al_2_O_3_ catalyst, the step (I) is adsorption of L-phenylalanine methyl ester onto the catalyst surface. The amino acid ester ad-species can be obtained by adsorption of amino group and ester group of L-phenylalanine methyl ester onto the sites of the catalyst surface. The active (or adsorption) sites on the γ-Al_2_O_3_(100) facet are greatly important for adsorption of amino group in the substrate molecules. The oxidic (O) sites are able to adsorb substrate molecules by adsorption of the amino group (–NH_2_) to form hydrogen bond by hydrogen (H_l_ and H_r_) of –NH_2_. Metallic Al sites are able to interact with the lone electron pair of nitrogen in amino group to form the coordination bond. The other active sites for interaction with the carbonyl group oxygen and alkoxy group oxygen of substrate are surface Al sites and Cu sites situated in the boundary between Cu cluster and Al_2_O_3_. These active sites are capable of interacting with the lone electron pair of the alkoxy group oxygen or carbonyl group oxygen.

The above DFT calculations demonstrate that the amino group bound on Al_2_O_3_ is an important contribution to enhancing the adsorption strength of the reactant, which are consistent with experimental results. For the reaction substrate without amino group substituent on the α-carbon, the catalytic hydrogenation hardly proceeded. Therefore, in the step (I), the preferential adsorption of amino group of substrate molecule may play a vital role in this catalytic cycle. In addition, the increasing electron-donor property of R group can increase an electron density around the alkoxy group oxygen atom, which is conducive to an increase of the adsorption energy of reactant and its reactivity.

**Figure 5:**
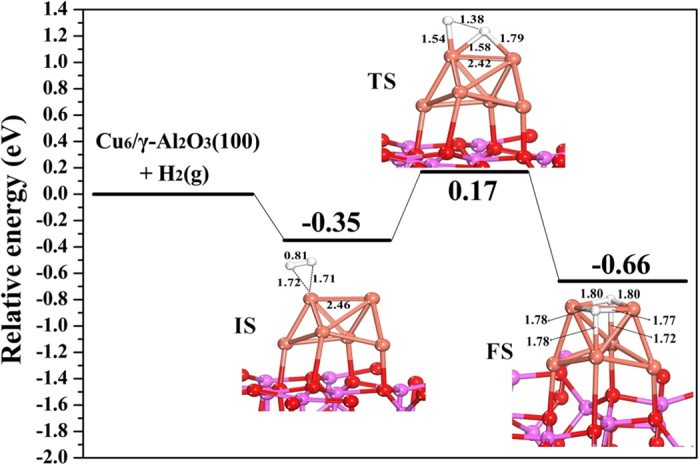
Potential energy profile and geometric structures of the initial state (IS), transition state (TS), and final state (FS) for H_2_ dissociation on the Cu_6_/γ-Al_2_O_3_(100) surface. (Bond lengths are reported in Å).

Step (II) is dissociation of hydrogen to activated H form (H_ad_). H_2_ can adsorb dissociatively on metallic copper to produce activated H species^31^,^32^. The presence of Zn and Mg in Cu/Al_2_O_3_ can promote the dispersion of Cu, which can help to adsorption and dissociation of hydrogen molecules^13^,^14^,^15^. The dissociative adsorption of H_2_ on the Cu_6_/γ-Al_2_O_3_(100) surface was studied, and the structures of its transition state (TS), final state (FS) and the potential energy profile are presented in Fig. 5. The results show that H_2_ adsorbed at the top Cu site is a precursor or initial state (IS) with a H–H bond length of 0.81 Å and adsorption energy of −0.35 eV. The activated H atom points toward the adjacent bridge site to form the transition state (TS) with the energy barrier of 0.52 eV, and the H–H bond is stretched to 1.38 Å, which suggests that H_2_ can relatively easily be dissociated to H atoms on the Cu surface. Finally, the dissociated H atoms adsorbed at the three-fold hollow sites on the Cu cluster, and the dissociation process is exothermic by −0.31 eV.

**Figure 6:**
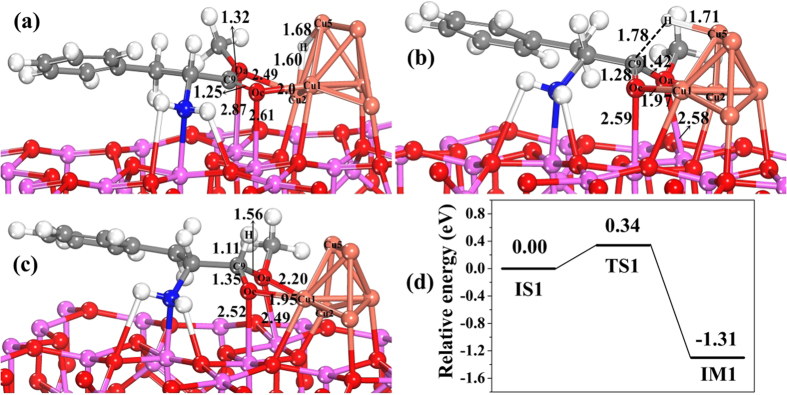
The structures and potential energy profile for H_ad_ transferring from Cu to C=O on Cu_6_/γ-Al_2_O_3_(100) in step III of the catalytic cycle. (**a**) Initial state of co-adsorbed L-p-me and H (IS1), (**b**) structure of transition state (TS1), (**c**) structure of intermediate (IM1), and (**d**) the corresponding potential energy profile. (Bond lengths are reported in Å).

Step (III) is that hydride attacks the carbonyl species. Compared with free molecule of L-p-me, the bond length of C_9_-O_a_ of adsorbed L-p-me is shorten from 1.36 to 1.32 Å (Table 5), indicating that it is hard to cleave the C_9_-O_a_ bond in adsorbed L-p-me. On the contrary, the bond length of C=O of adsorbed L-p-me is elongated from 1.22 (free molecule) to 1.25 Å, suggesting that the interaction between O_c_ atoms and the surface sites weakens the bond of C=O. This is similar to the situation reported that the combination of the surface active sites with the oxygen atom of C=O bond polarized the C=O bond of the substrate molecule (such as dimethyl adipate, methyl propionate and ethyl stearate) by the lone pair electron of the O atom, which is favorable for an attack of activated H to the carbon atom of the carbonyl group^33^,^34^,^35^,^36^. The activation energy barrier for H_ad_ transferring from Cu to C=O on the Cu_6_/γ-Al_2_O_3_(100) in this step was calculated, and its structures and corresponding potential energy profile are shown in Fig. 6. In the initial sate (IS1) (Fig. 6a), H_ad_ approaches the C_9_ atom of C=O, and then H_ad_ transfers from the Cu_2_-Cu_5_ bridge site to the C_9_ atom by the transition state (TS1), in which the distance between C_9_ and H is 1.78 Å, the bond length of Cu_5_-H is 1.71 Å, the bond lengths of C_9_-O_a_ and C_9_=O_c_ are stretched from 1.32 to 1.42 Å and from 1.25 to 1.28 Å, respectively. The O_a_-Al bond length decreases from 2.87 to 2.58 Å. The C_9_-H bond forms in a hemiacetal intermediate (IM1), in which the bond lengths of C_9_-O_a_ and C_9_=O_c_ are further stretched to 1.56 and 1.35 Å, respectively. In this step, the energy barrier is 0.34 eV and the process is exothermic by −1.31 eV (Fig. 6d), which shows that formation of hemiacetal intermediate on the Cu_6_/γ-Al_2_O_3_(100) surface is more favorable kinetically and thermodynamically. Therefore, forming hemiacetal intermediate by hydrogenation of adsorbed amino acid ester is the important step for this title reaction.

As shown in Table 5, compared with the adsorbed L-phenylalanine methyl ester, the bond length of O_a_-Al in adsorbed hemiacetal is shortened from 2.87 to 2.49 Å, and the bond length of O_a_-Cu_2_ is decreased from 2.49 to 2.20 Å, which suggests that the adsorption of alkoxy oxygen (O_a_) on the surface becomes stronger after the hemiacetal intermediate forms by hydrogenation of the adsorbed amino acid ester. The similar trends can be found in the O_c_-Al and O_c_-Cu_1_ bonds. Thus the adsorption energy of hemiacetal intermediate (−2.84 eV) is significantly higher than that of adsorbed amino acid ester (−1.18 eV). This result suggests that hemiacetal formation is thermodynamically feasible. In contrast, the bond length of C_9_-O_a_ is elongated from original 1.32 to 1.56 Å, indicating that the C_9_-O_a_ bond is largely weakened after hydrogenation of adsorbed amino acid ester. We infer that the weakening of C_9_-O_a_ bond makes easy cleavage of the C_9_-O_a_ bond for the next step.

**Figure 7:**
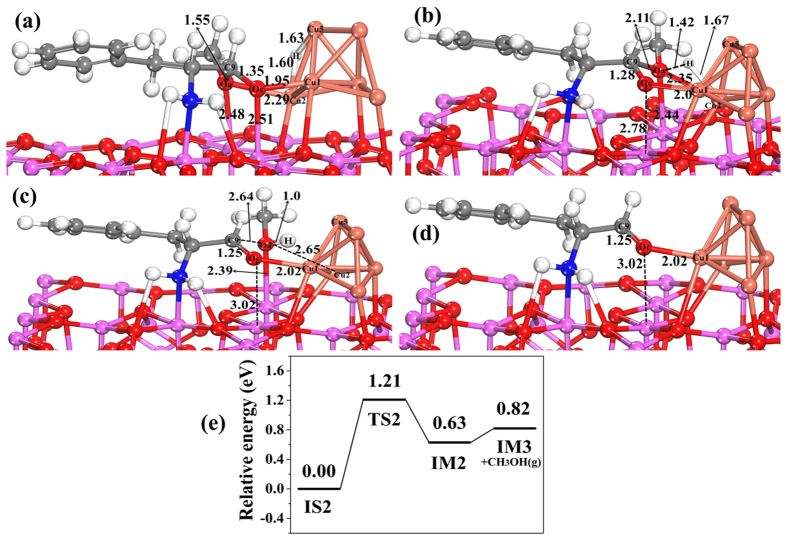
The structures and potential energy profile for the cleaving of C–O bond in hemiacetal by reacting with activated H on Cu_6_/γ-Al_2_O_3_(100) in step IV of the catalytic cycle. (**a**) Initial state of co-adsorbed hemiacetal and H (IS2), (**b**) structure of transition state (TS2), (**c**) structure of intermediate (IM2), (**d**) structure of intermediate (IM3) after desorption of CH_3_OH, and (**e**) the corresponding potential energy profile. (Bond lengths are reported in Å).

Step (IV) is the cleaving of C–O bond in the hemiacetal adsorbed species by reaction with activated H. The structures and corresponding potential energy profile for H_ad_ attacking hemiacetal ad-species and the cleaving of C–O bond in hemiacetal by reacting with H_ad_ on Cu_6_/γ-Al_2_O_3_(100) are shown in Fig. 7. In the initial state of co-adsorbed hemiacetal and H (IS2), H_ad_ approaches the O_a_ atom in hemiacetal, and then H_ad_ migrates out of the Cu_2_ and Cu_5_ atoms to bond the O_a_ atom of methoxyl, which forms the transition state (TS2, Fig. 7b), followed by breaking the C_9_-O_a_ bond and Cu_2_-O_a_ bond to form the a O_a_-H bond, and its intermediate structure (IM2) is shown in Fig. 7c. In TS2, the distance between H_ad_ and O_a_ decreases to 1.42 Å, the bond length of Cu_2_-H is 1.67 Å, and the bond lengths of C_9_-O_a_ and O_c_-Al are further stretched from 1.55 to 2.11 Å and from 2.51 to 2.78 Å, respectively. And the bond length of C_9_-O_c_ decreases from 1.35 to 1.28 Å. This step is endothermic by 0.63 eV with the relatively high energy barrier of 1.21 eV (Fig. 7e), which is likely to the rate-determining step in this title reaction. After the C–O bond in hemiacetal intermediate splits to the amino aldehyde and methanol ad-species, the distance between O_a_ and Cu_2_ increases from 2.29 (in IS2) to 2.65 Å, and the distance between O_c_ and Al increases from 2.51 (in IS2) to 3.02 Å, indicating that the adsorption strength of O_a_ in CH_3_OH on the surface becomes weaker, and the corresponding adsorption energy of adsorbed amino aldehyde and methanol is lower (−1.23 eV, Table 5). Therefore, CH_3_OH is easily released to the gas phase with a small desorption energy of 0.19 eV (Fig. 7e), and amino aldehyde intermediate (IM3) resides on the Cu_6_/γ-Al_2_O_3_(100) surface (Fig. 7d). The calculated adsorption energy of amino aldehyde intermediate (IM3) is −1.02 eV (Table 5).

Thus, in this step, the dissociated H attacks against the hemiacetal ad-species by cleavage of the C–O bond to generate the corresponding amino aldehyde and methanol ad-species. Subsequently, methanol desorbs from the surface.

**Figure 8:**
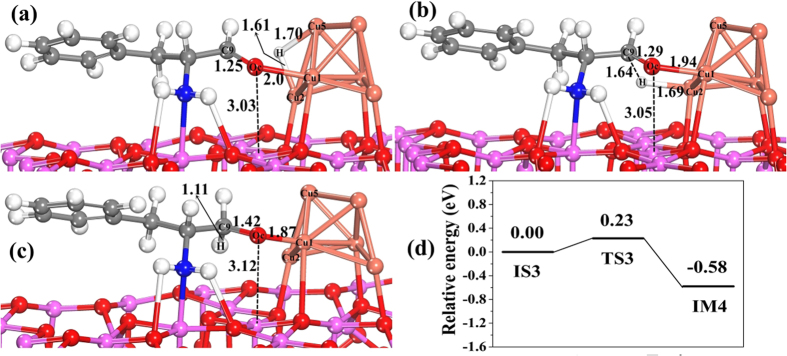
The structures and potential energy profile for H_ad_ attacking C of C=O in amino aldehyde on Cu_6_/γ-Al_2_O_3_(100) in step V of the catalytic cycle. (**a**) Initial state of co-adsorbed amino aldehyde and H (IS3), (**b**) structure of transition state (TS3), (**c**) structure of intermediate (IM4), and (**d**) the corresponding potential energy profile. (Bond lengths are reported in Å).

**Figure 9:**
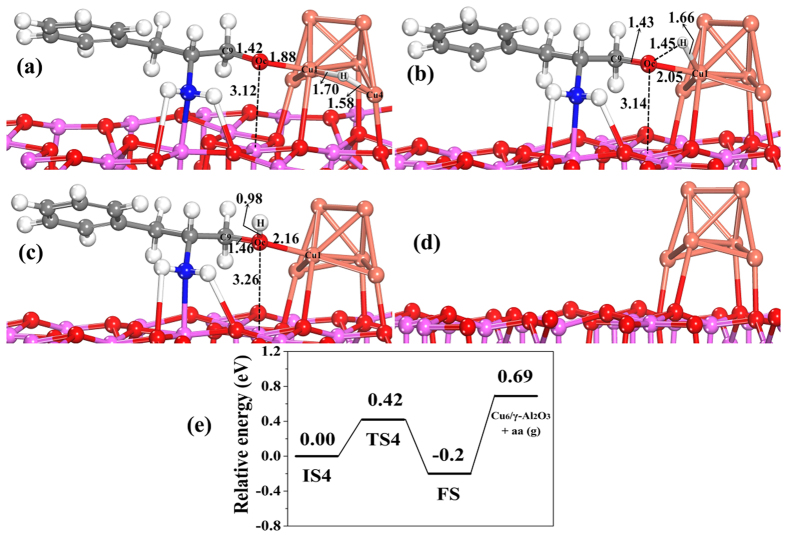
The structures and potential energy profile for H_ad_ attacking O_ad_ species on Cu_6_/γ-Al_2_O_3_(100) in step V of the catalytic cycle. (**a**) Initial state of co-adsorbed IM4 and H (IS4), (**b**) structure of transition state (TS4), (**c**) structure of final state (FS), (**d**) structure of Cu_6_/γ-Al_2_O_3_(100) after desorption of amino alcohol (aa), and (**e**) the corresponding potential energy profile. (Bond lengths are reported in Å).

Step (V) is hydrogenation of amino aldehyde ad-species. This step involves two elementary reactions, and their structures of the initial state, transition state, intermediate and product are shown in Figs 8 and 9, in which the potential energy profiles are included. As shown in Fig. 8, after H_ad_ attacks C_9_ in C=O, H_ad_ shifts from the Cu_2_-Cu_5_ bridge sites to C_9_ of adsorbed amino aldehyde by TS3 (Fig. 8b). In TS3, the bond length of C_9_=O_c_ is elongated from 1.25 to 1.29 Å, and the distance between C_9_ and H is 1.64 Å, followed by the formation of the C_9_-H bond in intermediate IM4. This first elementary reaction is exothermic by −0.58 eV, with a relatively small activation barrier of 0.23 eV (Fig. 8d).

Then, the adsorbed speices in IS4 (Fig. 9a) convert into amino alcohol (aa) product in FS by the transition state (TS4) (Fig. 9b,c). This step is exothermic by −0.2 eV, and the energy barrier is 0.42 eV. These results indicate that amino aldehyde is relatively easily hydrogenated to an amino alcohol. As shown in Fig. 9c for the adsorption configurations of amino alcohol, the amino group is bonded with the O-Al-O sites on the surface. The O_c_ atom interacts with Cu sites. The calculated adsorption energy of the amino alcohol on Cu_6_/γ-Al_2_O_3_(100) is −0.89 eV (Table 5), which shows that amino alcohol is easy to desorb from the catalyst surface.

Step (VI) is desorption of amino alcohol adsorbed on Cu_6_/γ-Al_2_O_3_(100) from the catalyst surface, and recovery of the catalyst active sites. After the amino alcohol product desorbs from the catalyst surface with a desorption energy of 0.89 eV (Fig. 9c,e), and the catalyst active sites could be recovered (Fig. 9d).

### The role of amino group in the substrate

**Figure 10:**
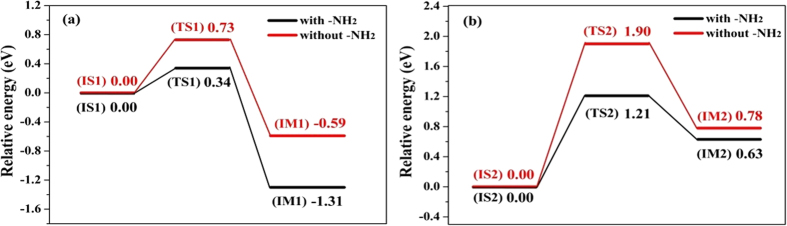
The potential energy profiles for hydrogenation of L-p-me (black line) and 3-p-a-me (red line). (**a**) H_ad_ transferring from Cu to C=O on Cu_6_/γ-Al_2_O_3_(100) in step III of the catalytic cycle, and (**b**) the cleaving of C–O bond in hemiacetal by reacting with activated H on Cu_6_/γ-Al_2_O_3_(100) in step IV of the catalytic cycle.

To gain more insight into the role of the amino group of L-phenylalanine methyl ester (L-p-me) in the L-p-me hydrogenation over the Cu_6_/γ-Al_2_O_3_(100) catalyst, the energy barriers for the transferring of H_ad_ from Cu to C=O (Step III) and the cleaving of C–O bond in hemiacetal (Step IV, rate-determining step) were calculated for 3-phenylpropionic acid methyl ester (3-p-a-me) and L-p-me as the substrates. The potential energy profiles for hydrogenation of L-p-me and 3-p-a-me are shown in Fig. 10, and their corresponding structures of initial states, transition states and intermediates are given in Fig. S6. As shown in Fig. 10, in hydrogenation of the substrate without amino group, the energy barriers for step III and step IV (rate-limiting step) are significantly higher than these in the L-p-me hydrogenation, which is consistent with the experimental results that a high reaction temperature (>240 °C) is usually needed for the effective transformation of alkyl carboxylic esters to the corresponding alcohols over the Cu-based catalysts^31^,^37^,^38^,^39^,^40^, and hydrogenation of 3-p-a-me at 110 °C was not effective over this CuZn_0.3_Mg_0.1_AlO_x_ catalyst (Table 1).

Therefore, it is reasonable to conclude that the adsorption of amino group in the substrate plays a vital role in this catalytic hydrogenation cycle, which not only activates the substrate and stabilizes the adsorption species to start up whole catalytic hydrogenation reaction, but also reduces the activation energy barrier for this title reaction.

## Conclusions

In summary, the effects of L-phenylalanine esters with different ester group substituents and protection of amino group as the substrates on the catalytic hydrogenation over the CuZn_0.3_Mg_0.1_AlO_x_ catalyst were investigated, verifying that the CuZn_0.3_Mg_0.1_AlO_x_ catalyst is an effective heterogeneous catalyst for the hydrogenation of amino acid esters to chiral amino alcohols. To identify the preferred active sites on the catalyst for adsorbing functional groups of substrates and explore the possible reaction pathways for hydrogenation of L-phenylalanine methyl ester to L-phenylalaninol, the DFT theoretical calculations for substrates adsorption on the Cu_6_/γ-Al_2_O_3_(100) model were also performed. The results show that the presence of amino group at the α-position of carboxy group in α-amino acid esters is essential for this catalytic hydrogenation. The first adsorption of the amino group on the catalyst surface sites is very important for activating substrate to start up whole catalytic hydrogenation. The different substituents in amino acid esters have a remarkable influence on the adsorption of alkoxy group oxygen and carbonyl group oxygen, and the catalytic reactivity increases in the order of the substrates with the substituent of CH_2_CF_3_ < CH_2_Cl < CH_2_CH_3_ ≈ CH_3_. When the electron-donor ability of the ester group substituent is increased, its adsorption energy increases and the dissociation activation energy of ester group decreases, resulting in the increase in the substrate reactivity. When the ester group substituent is the group of t-Bu or benzyl, the distance between alkoxy group oxygen atoms and the active sites on the catalyst surface is elongated, due to the steric-hindrance effect and a decrease in the adsorption energy, thus leading to the lower reactivity of L-phenylalanine t-Bu ester and L-phenylalanine benzyl ester.

This catalytic hydrogenation reaction undergoes the formation of a hemiacetal intermediate, the cleavage of the C–O bond by reacting with dissociated H, followed by the formation of the amino aldehyde and methanol ad-species, which is the rate-determining step. Then methanol desorbs from the catalyst surface, and aldehyde ad-species is instantly hydrogenated to the desired amino alcohol. The CuZn_0.3_Mg_0.1_AlO_x_ catalyst can be applied in the hydrogenation of various α-amino acid esters, and the α-amino acid esters with small ester group substituents containing the electron-donor properties should be the most active substrates.

## Experimental section

### Catalyst preparation

The CuZn_0.3_Mg_0.1_AlO_x_ catalyst was prepared by the co-precipitated method with Cu(NO_3_)_2_∙3H_2_O, Zn(NO_3_)_2_∙6H_2_O, Al(NO_3_)_3_∙9H_2_O, Mg(NO_3_)_2_∙6H_2_O and Na_2_CO_3_ (A.R., Sinopharm Chemical Reagent Co. Ltd.), and the preparation procedure was described as follows. 1.0 M Cu(NO_3_)_2_, 1.0 M Zn(NO_3_)_2_, 1.0 M Al(NO_3_)_3_, 1.0 M Mg(NO_3_)_2_ and 0.5 M Na_2_CO_3_ aqueous solutions were prepared, respectively. The mixture solution of Cu^2+^ and Zn^2+^ and the 0.5 M Na_2_CO_3_ solution were co-precipitated in one pot at 70 °C under vigorous stirring. During the precipitation process, the flow rates of two solutions were adjusted to keep a constant pH value of 7.5. And the mixture solution of Mg^2+^ and Al^3+^ and 0.5 M Na_2_CO_3_ solution were co-precipitated in another pot under the same condition above. Then two solutions obtained above were mixed, and then this synthesis solution was aged under vigorous stirring for 2 h and cooled statically for 1 h. After filtrating and washing with deionized water (70 °C) until the filtrate was neutral, the synthesized precursor material was dried at room temperature for 12 h, then dried at 120 °C for 24 h, and calcined at 450 °C for 4 h at a heating rate of 5 °C/min. Finally, the calcined sample was pressed, crushed, and sieved to 0.45–0.85 mm (20–40 mesh). In addition, the Cu_a_Zn_b_Mg_c_Al_d_O_y_ catalysts with different metal mole ratio (Table S1) were prepared by the same as above preparation procedure except for the contents of metal nitrates.

### Preparation of the reaction substrates

3-phenylpropionic acid methyl ester (99%, Adamas), L-phenylalanine (99%, Adamas) and N-Boc-L-phenylalanine methyl ester (98%, Adamas) were used directly. The amino acid esters, except for L-phenylalanine trifluoroethyl ester and L-phenylalanine chloromethyl ester, were obtained from their hydrochloride (98%, Adamas). For example, L-phenylalanine methyl ester was prepared from methyl L-phenylalaninate hydrochloride by the reaction formula Eq. (1). Methyl L-phenylalaninate hydrochloride was dissolved in de-ionized water, and then adjusted pH to ~8 by adding sodium carbonate solution. After extraction with ethyl acetate, the extract was dried by anhydrous magnesium sulfate for 30 min. After filtration, L-phenylalanine methyl ester was obtained by rotary evaporation.


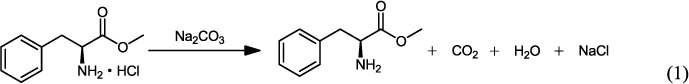


L-phenylalanine trifluoroethyl ester and L-phenylalanine chloromethyl ester were obtained from N-Boc-L-phenylalanine trifluoroethyl ester and N-Boc-L-phenylalanine chloromethyl ester (Shanghai Synteam Biochem CO., Ltd), and their structures were determined by ^1^H NMR (Figs S7 and S8). The preparation procedure was as follows. N-Boc-L-phenylalanine trifluoroethyl ester (or N-Boc-L-phenylalanine chloromethyl ester) was dissolved in the ice-cooled and saturated solution of hydrogen chloride in ethyl acetate. The mixture was stirred at room temperature for 2 h, and then the reaction mixture was washed with Na_2_CO_3_ solution. The organic phase was dried with MgSO_4_, filtered and evaporated to obtain L-phenylalanine trifluoroethyl ester (or L-Phenylalanine chloromethyl ester).

### Catalytic activity testing

The catalytic activity of the catalyst for the title hydrogenation was tested in a 500 mL stainless steel autoclave under stirring at a speed of 500 rpm. After 1.0 g catalyst (20–40 mesh) was added into the reactor, this reactor was purged with 4 MPa H_2_ to expel air 4 times and the catalyst was reduced in 1 MPa H_2_ at 250 °C for 4 h, and then the reactor was cooled to ambient temperature. 1.5 g substrate diluted in 150 mL ethanol (the exception is that 1.5 g L-phenylalanine was dissolved in mixture of water/ethanol (2/1, volume) by ultrasound dispersion) was introduced, in which *Substrate*/*Cat.* = 1.5 (mass ratio). The typical reaction conditions were 4 MPa of H_2_ and 110 °C. After the reaction was completed, the reactor was cooled to room temperature, and the pressure was released. The catalyst was separated by centrifugation, and the products were analyzed by HPLC (High Performance Liquid Chromatography), and ^1^H NMR (Bruker, AVANCE III 500 MHz).

HPLC analysis was done on an Agilent 1260 Infinity equipped with an ultraviolet detector (wavelength 254 nm) and a column (Poroshell 120 EC-C18, 50 × 4.6 mm, 2.7 μm particle size). The mobile phase was 0.05 mol/L ammonium acetate aqueous solution (pH = 5.0) containing 5–25 vol.% methanol for different reaction substrates (for N-Boc-L-phenylalanine methyl ester and 3-phenylpropionic acid methyl ester, the mobile phase was water/methanol = 1/1 (vol) without ammonium acetate) and its flow rate was 0.6 mL/min, and column temperature was 35 °C. Experimental errors for the conversion and selectivity are within ±2%.

The conversion of reaction substrate (*X*) = (Mass of reaction substrate converted/Total mass of reaction substrate in the feed) × 100%;

The L-phenylalaninol chemselectivity (*S*) = (Mass of L-phenylalaninol formed actually/Mass of L-phenylalaninol formed theoretically in *X* conversion) × 100%;

The L-phenylalaninol yield (*Y*) = *X* × *S* × 100%.

The enantiomeric excess (*ee*) of phenylalanine esters and phenylalaninol was determined by HPLC (Agilent 1260 Infinity) equipped with an ultraviolet detector (wavelength 258 nm) and a chiral column (CHIRALPAK AY-H, 250 × 4.6 mm, 5 μm particle size). The mobile phase was n-Hexane/EtOH/Ethanolamine = 90/10/0.1 (vol.) and its flow rate was 1 mL/min, and column temperature was 35 °C.

*ee* (%) of phenylalaninol = (CPA of L-phenylalaninol − CPA of D-phenylalaninol)/(CPA of L-phenylalaninol + CPA of D-phenylalaninol), where CPA is the chromatographic peak area.

### The structure and *ee* value of product

^1^H NMR spectrum of product L-phenylalaninol was obtained on a Bruker AVANCE III 500 and shown in Fig. S9. ^1^H NMR (CDCl_3_): 7.21~7.35 (5H, m, Ph-H), 3.42~3.68 (2H, m, -CH_2_-O-), 3.15 (1H, s, -CH-N-), 2.53~2.84 (2H, m, CH_2_-Ph), 1.83 (2H, b, -NH_2_). For all the phenylalaninol synthetized by different reaction substrates, their chiral HPLC chromatograms were shown in Figs S10, S11, S12, S13, S14, S15, S16 and S17. The results show that only L-phenylalaninol could be detected, that is to say, their *ee* value is ~100% and the chiralities of reactants can be well maintained after the reaction at 110 °C and 4 MPa of H_2_.

### Computational details of DFT study

To identify the preferred adsorption sites over the catalyst for the functional groups of substrates, theoretical calculations were performed by a density-functional theory (DFT) with the Perdew-Wang 91 generalized gradient approximation (GGA-PW91) by using the VASP code^41^,^42^, in which GGA was adopted to solve the Kohn-Sham equations. We employed the project-augmented wave (PAW) method to represent the core–valence interaction^43^. The valence electronic states were expanded in plane-wave basis sets with an energy cut-off at 450 eV.

In the DFT calculation, Cu_6_/γ-Al_2_O_3_(100) was used instead of CuZn_0.3_Mg_0.1_AlO_x_ as the model catalyst, and the reason is as follows. The composition of CuZn_0.3_Mg_0.1_AlO_x_ is too complicated for the DFT calculation. Our previous research results show that^13^,^14^,^15^, the role of doping Zn and Mg in CuO/Al_2_O_3_ is to improve the dispersion of Cu, which is conducive to adsorption and dissociation of H_2_. To prove the roles of different metal components in the catalyst, we designed a series of Cu_a_Zn_b_Mg_c_Al_d_O_y_ samples with different compositions, and their catalytic activities for L-phenylalaninol synthesis are listed in Table S1. The results show that a little selectivity of L-phenylalaninol was obtained on Zn_0.3_AlO_y_ (without Cu) and CuZn_0.3_O_y_ (without Al). Using the CuAlO_y_ catalyst, 75.6% yield of L-phenylalaninol can be achieved, and the doping Zn and Mg can further improve the yield. Based on the above results, we can verify that Cu and Al are the main active component in the CuZn_0.3_Mg_0.1_AlO_x_ catalyst, thus we chose Cu/Al_2_O_3_ as the model catalyst.

The crystallographic data of γ-Al_2_O_3_ bulk structure was adopted on the basis of the model reported by Digne *et al*.^44^: the optimized lattice parameters of γ-Al_2_O_3_ were taken as a = 5.57, b = 8.39, c = 8.05 Å, and β = 90.59°. There are mainly the (110), (100) and (111) facets in γ-Al_2_O_3_^44^. To our knowledge, in the theoretical calculation, the (111) facet of γ-Al_2_O_3_ was lesser adopted, probably due to its higher surface energy and unstable, because the (111) facet is only ~10% of the total area^44^. Therefore, most of the DFT studies focus on the (110) or (100) surface of γ-Al_2_O_3_. For instance, in the CO_2_ hydrogenation over Cu/γ-Al_2_O_3_^45^, Pd/γ-Al_2_O_3_^46^ and Ni/γ-Al_2_O_3_^47^, the (110) facet of γ-Al_2_O_3_ was used as the theoretical calculation model. And the (100) facet of γ-Al_2_O_3_ was widely used, such as, the preparation of ethylene and diethyl ether from ethanol^48^, CH_4_ and H_2_ dissociation on Ni/γ-Al_2_O_3_(100)^49^, 2-butanol dehydration on γ-Al_2_O_3_(100)^50^, and the reactions mechanism of 2-butanol over the γ-alumina (100) surface^51^. We have studied the adsorption of substrate on the γ-Al_2_O_3_(110) facet by DFT calculation, but we found that it is less favorable compared with the adsorption of substrate on the γ-Al_2_O_3_(100) facet. Associated with the experimental results, we thought that the γ-Al_2_O_3_(100) facet should be the appropriate active surface rather than the (110) facet of γ-Al_2_O_3_. Not only the (100) surface of γ-Al_2_O_3_ has been widely studied^48^,^49^,^50^,^51^, but also the γ-Al_2_O_3_(100) surface is one of the most discovered and catalytically active surfaces for anchoring deposited transition metal and metal oxide particles^49^,^52^. And its (100) surface exposes the penta-coordinated Al sites that were observed experimentally^52^,^53^,^54^. Based on the reasons above, we have adopted the γ-Al_2_O_3_(100) facet as the calculation model. In the γ-Al_2_O_3_(100) model, a 6-layer slab model contained 48 Al_2_O_3_ units and the vacuum region between the slabs was set to 16 Å. The transition states (TS) structures were searched by using Constrained Broyden Minimization method^55^. The force threshold for the optimization and transition state search was 0.05 eV Å^−1^.

It was reported that the 4-atom Cu cluster on γ-Al_2_O_3_ surface was employed to study CO_2_ hydrogenation^45^ and glycerol hydrogenolysis^56^. This Cu_4_ cluster is the smallest unit which can provide a three-dimensional structure to probe both metal-metal and metal-support interactions. The adsorption energy of L-phenylalanine methyl ester adsorbed on Cu_4_/γ-Al_2_O_3_(100) was calculated to be −0.91 eV, and the distances between O_a_ and Cu_1_, O_a_ and Al are 3.11 Å and 3.21 Å, respectively (Fig. S18). These results indicate that the Cu_4_ cluster model was inappropriate for the adsorption of this large molecule. Therefore, we adopted the larger 6-atom Cu cluster supported on the γ-Al_2_O_3_(100) facet as the model catalyst. Only the bottom four atomic layers of the γ-Al_2_O_3_(100) facet are frozen in their bulk positions, whereas the top two atomic layers of the slab together with the Cu_6_ cluster and the adsorbates are allowed to relax. A *p*(2 × 2) surface cell with 1 × 1 × 1 *k*-point mesh with an area of 11.17 × 16.83 Å^2^ was used to fully take the relaxation effects into account. In this work, the adsorption energies of molecules (*e.g.*, L-phenylalanine methyl ester) on Cu_6_/γ-Al_2_O_3_ were calculated as follow Eq. (2):


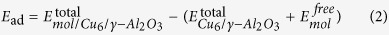


where 
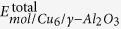
 represents the total energy of adsorbed molecules (mol) on the Cu_6_/γ-Al_2_O_3_ surface in the equilibrium state and 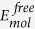 is the total energy of molecules in the gas-phase. The negative value of *E*_ad_ indicates an energy gain during adsorption.

## Additional Information

**How to cite this article**: Zhang, S. *et al*. High-effective approach from amino acid esters to chiral amino alcohols over Cu/ZnO/Al_2_O_3_ catalyst and its catalytic reaction mechanism. *Sci. Rep.* **6**, 33196; doi: 10.1038/srep33196 (2016).

## Supplementary Material

Supplementary Information

## Figures and Tables

**Table 1:**
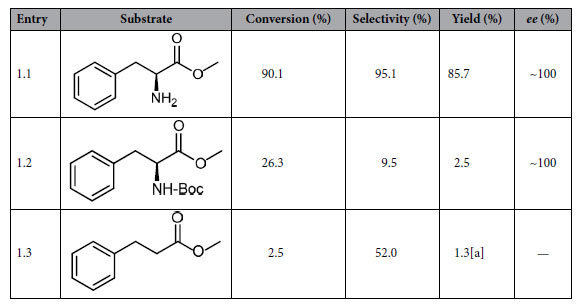
Effect of the amino group of substrate on the catalytic hydrogenation over the CuZn_0.3_Mg_0.1_AlO_x_ catalyst.

Reaction conditions: 1.0 g catalyst, 1.5 g reaction substrate, anhydrous ethanol (150 ml) as solvent, 4 MPa hydrogen pressure, 3 h of reaction at 110 °C.^[a]^Yield of 3-phenyl propyl alcohol.

**Table 2:**
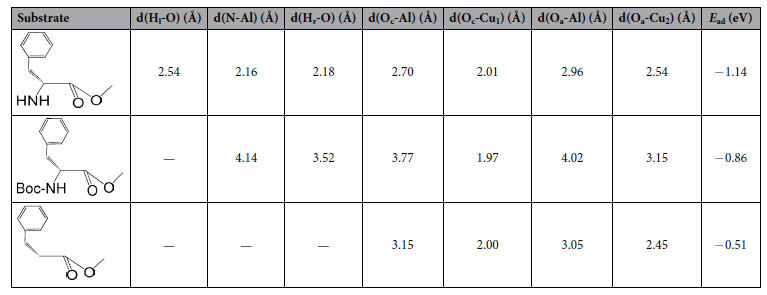
Structural parameters and adsorption energies of L-phenylalanine esters or 3-phenylpropionic acid methyl ester on Cu_6_/γ-Al_2_O_3_(100).

H_l_ and H_r_ represent the left H atom and right H atom of amino group respectively. d(H_l_-O) means the distance between H_l_ atoms and O atoms, and the other is the same as above.

**Table 3:**
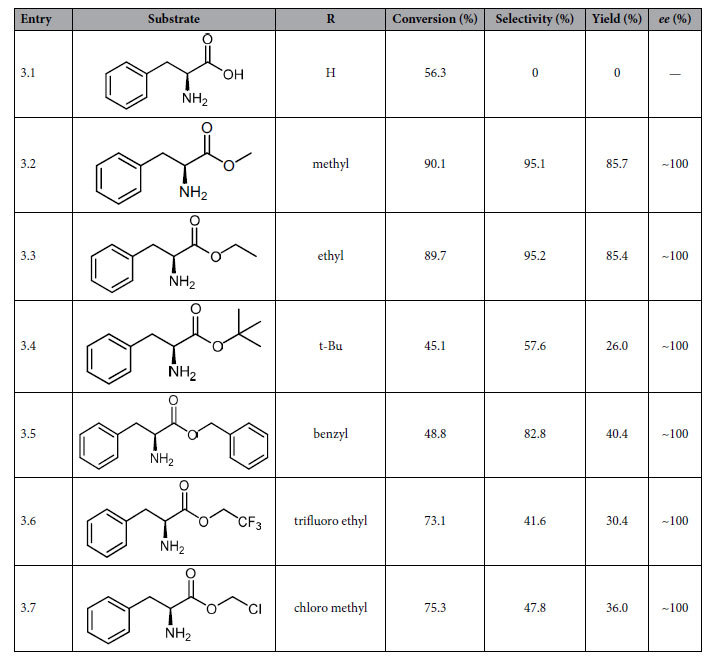
Effect of the ester group substituent in substrate on the catalytic hydrogenation over the CuZn_0.3_Mg_0.1_AlO_x_ catalyst.

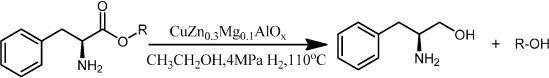
 Reaction conditions: 1.0 g CuZn_0.3_Mg_0.1_AlO_x_ catalyst, 1.5 g reaction substrate, anhydrous ethanol (150 ml) as solvent (except for L-phenylalanine), 4 MPa hydrogen pressure, 3 h of reaction at 110 °C.

**Table 4:**
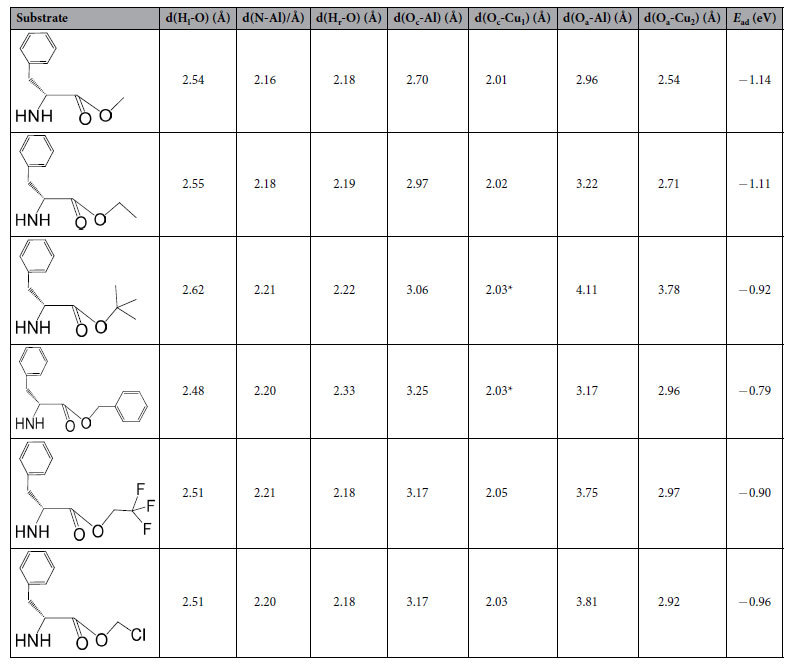
Structural parameters and adsorption energies of L-phenylalanine esters on Cu_6_/γ-Al_2_O_3_(100).

^*^Represents the distance of O_c_-Cu_2_, namely, d(O_c_-Cu_2_)(Å).

**Table 5:**
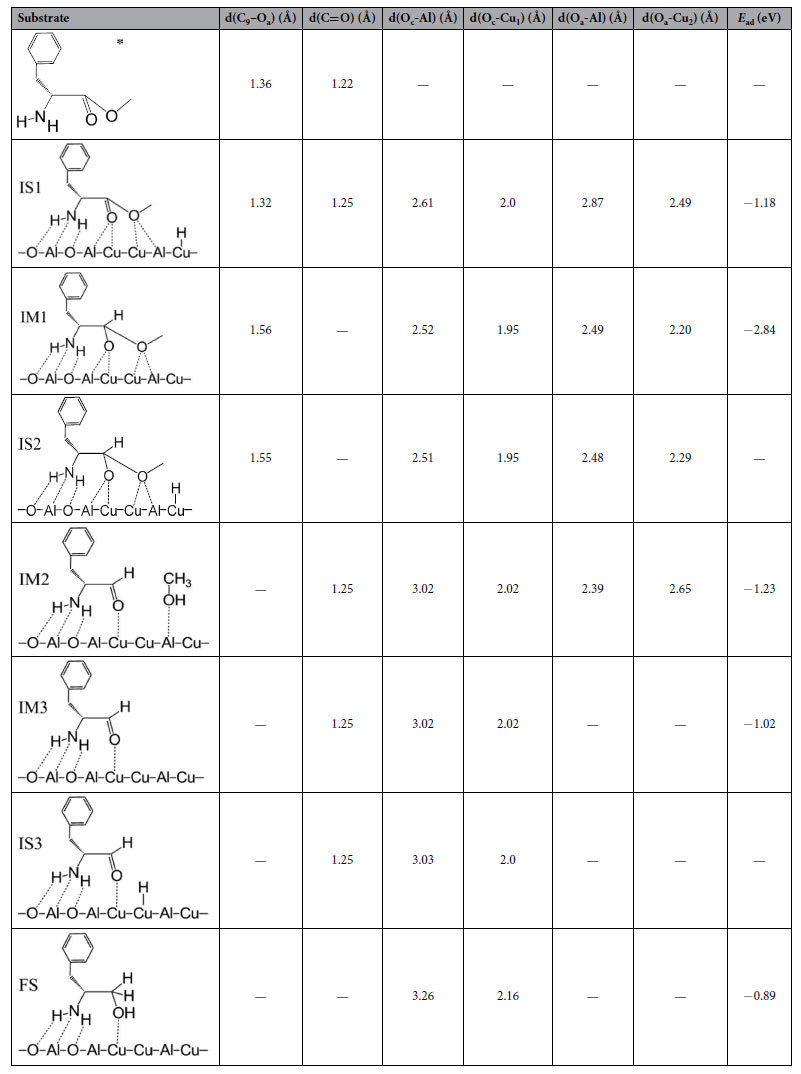
Structural parameters and adsorption energies of reactant on Cu_6_/γ-Al_2_O_3_(100) in the catalytic cycle.

^*^Represents the free molecule of L-p-me.
