# Supplementary material for: High-effective approach from amino acid esters to chiral amino alcohols over Cu/ZnO/Al2O3 catalyst and its catalytic reaction mechanism
Source: Sci Rep. 2016 Sep 13;6:33196. doi: 10.1038/srep33196 (PMC5020414; doi:10.1038/srep33196)
Supplement: Supplementary Information [file srep33196-s1.doc]

**Supplementary Information:**

**High-effective approach from** **amino acid esters to chiral amino alcohols over Cu/ZnO/Al_2_O_3_ catalyst and** **its catalytic reaction mechanism**

Shuangshuang Zhang,^1^ Jun Yu,^1^ Huiying Li,^1^ Dongsen Mao^1^ and Guanzhong Lu^1,2^*

^1^ Research Institute of Applied Catalysis, School of Chemical and Environmental Engineering, Shanghai Institute of Technology, Shanghai 201418, China.

^2^ Key Laboratory for Advanced Materials and Research Institute of Industrial catalysis, East China University of Science and Technology, Shanghai 200237, China.

*Corresponding Author: Fax: +86-21-64252923. E-mail: [gzhlu@ecust.edu.cn](mailto:gzhlu@ecust.edu.cn) (G.Z. Lu).

**Table S1.** Catalytic performance of Cu_a_Zn_b_Mg_c_Al_d_O_y_ for L-phenylalaninol synthesis at 110 °C for 5 h.

| Catalyst | Conversion^[a]^  (%) | Yield^[a]^  (%) | Selectivity  (%) |
| --- | --- | --- | --- |
| Cu_1_Zn_0.3_Mg_0_Al_0_O_y_ | 98.7 | 1.2 | 1.2 |
| Cu_0_Zn_0.3_Mg_0_Al_1_O_y_ | 100 | 0 | 0 |
| Cu_1_Zn_0_Mg_0_Al_1_O_y_ | 100 | 75.6 | 75.6 |
| Cu_1_Zn_0.3_Mg_0_Al_1_O_y_ | 100 | 82.4 | 82.4 |
| Cu_1_Zn_0.3_Mg_0.1_Al_1_O_y_ | 100 | 92.1 | 92.1 |
| **−** | 61.5 | 0 | 0 |

Reaction conditions: 1.0 g catalyst, 1.5 g L-phenylalanine methyl ester, anhydrous ethanol (150 ml) as solvent, 4 MPa hydrogen pressure.

^[a]^ Conversions and yields were determined by HPLC and ^1^H NMR.

As shown in Table S1, no selectivity of L-phenylalaninol was obtained without adding catalyst. The yield of L-phenylalaninol was only 1.2 % over the Cu_1_Zn_0.3_Mg_0_Al_0_O_y_ (CuO/ZnO) catalyst. Using the Cu_1_Zn_0_Mg_0_Al_1_O_y_ (CuO/Al_2_O_3_) catalyst, 75.6 % yield to L-phenylalaninol can be obtained, which shows that Al_2_O_3_ in Cu-based catalyst is very important for L-phenylalaninol synthesis. When the catalyst without CuO or Cu (e.g. Cu_0_Zn_0.3_Mg_0_Al_1_O_y_, that is ZnO/Al_2_O_3_) was used, a trace of desired product was not detected, which also indicates that Cu is a main active site for the title reaction. After adding Zn or Mg in CuO/Al_2_O_3_ (that is Cu_1_Zn_0_Mg_0_Al_1_O_y_), namely Cu_1_Zn_0.3_Mg_0_Al_1_O_y_ or Cu_1_Zn_0.3_Mg_0.1_Al_1_O_y_ catalyst, the L-phenylalaninol yield was increased, indicating that doping Zn and Mg can improve the dispersion of Cu and be in favor of adsorption and dissociation of hydrogen molecules.


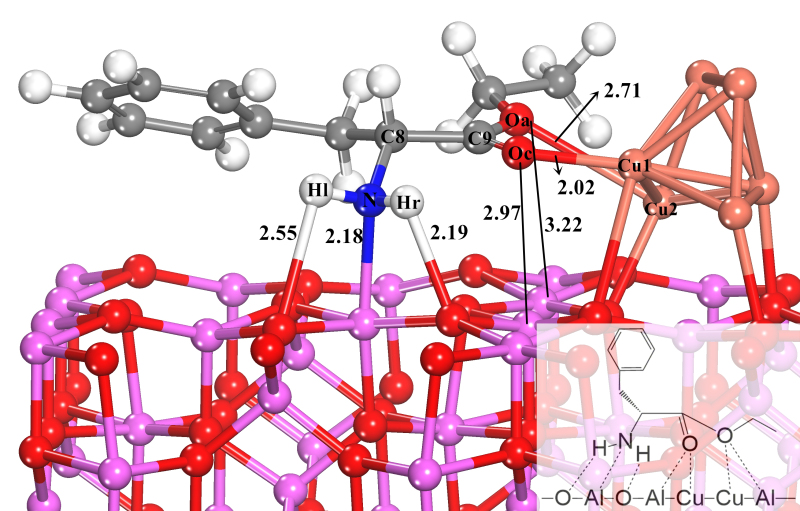


**Figure S1.** Optimized configuration of L-phenylalanine ethyl ester adsorbed on the Cu_6_/γ-Al_2_O_3_ (100) facet. The unit of distance between two atoms is Å.


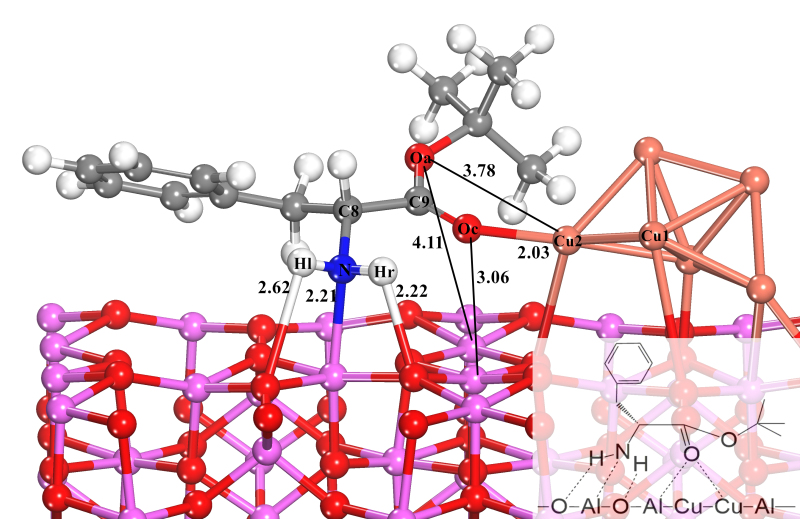


**Figure S2.** Optimized configuration of L-phenylalanine t-butyl ester adsorbed on the Cu_6_/γ-Al_2_O_3_ (100) facet. The unit of distance between two atoms is Å.


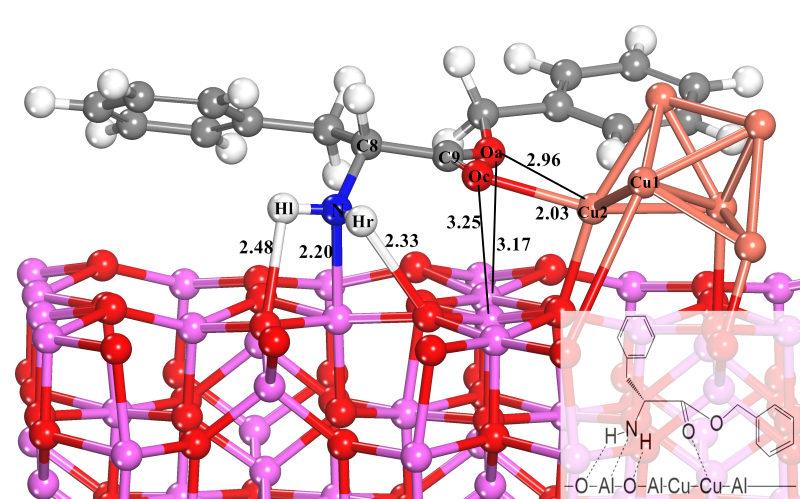


**Figure S3.** Optimized configuration of L-phenylalanine benzyl ester adsorbed on the Cu_6_/γ-Al_2_O_3_ (100) facet. The unit of distance between two atoms is Å.


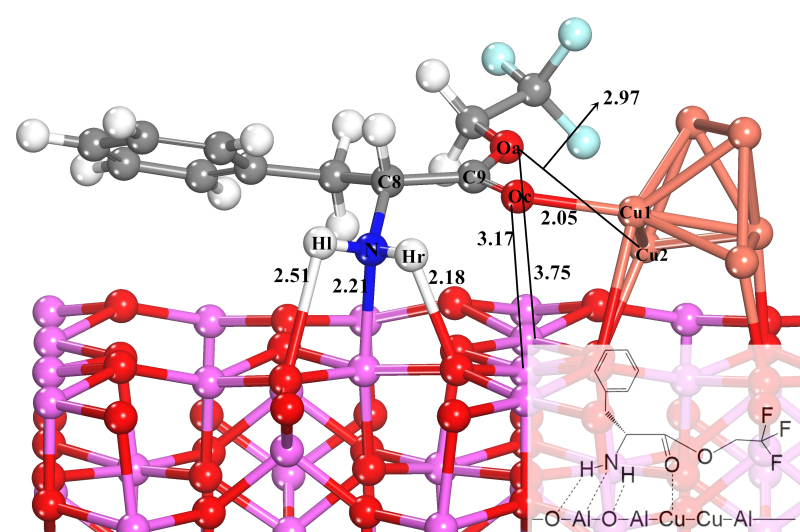


**Figure S4.** Optimized configuration of L-phenylalanine trifluoroethyl ester adsorbed on the Cu_6_/γ-Al_2_O_3_ (100) facet. F atoms are light-blue. The unit of distance between two atoms is Å.


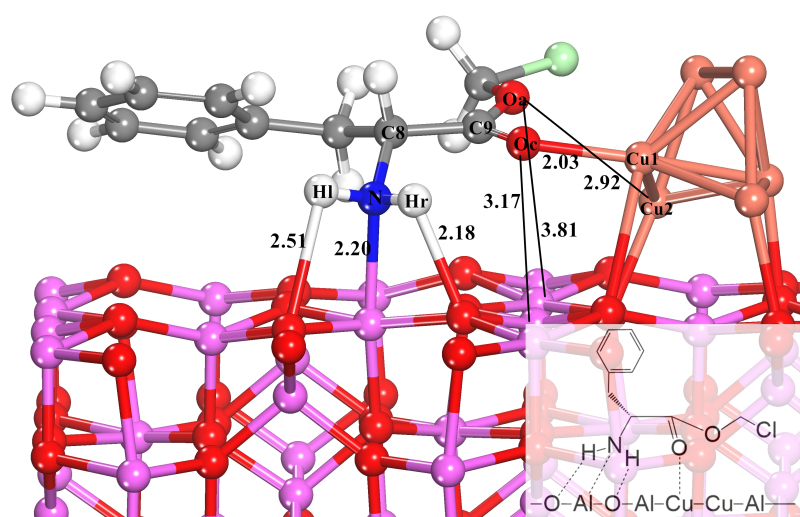


**Figure S5.** Optimized configuration of L-phenylalanine chloromethyl ester adsorbed on the Cu_6_/γ-Al_2_O_3_ (100) facet. Cl atoms are green. The unit of distance between two atoms is Å.


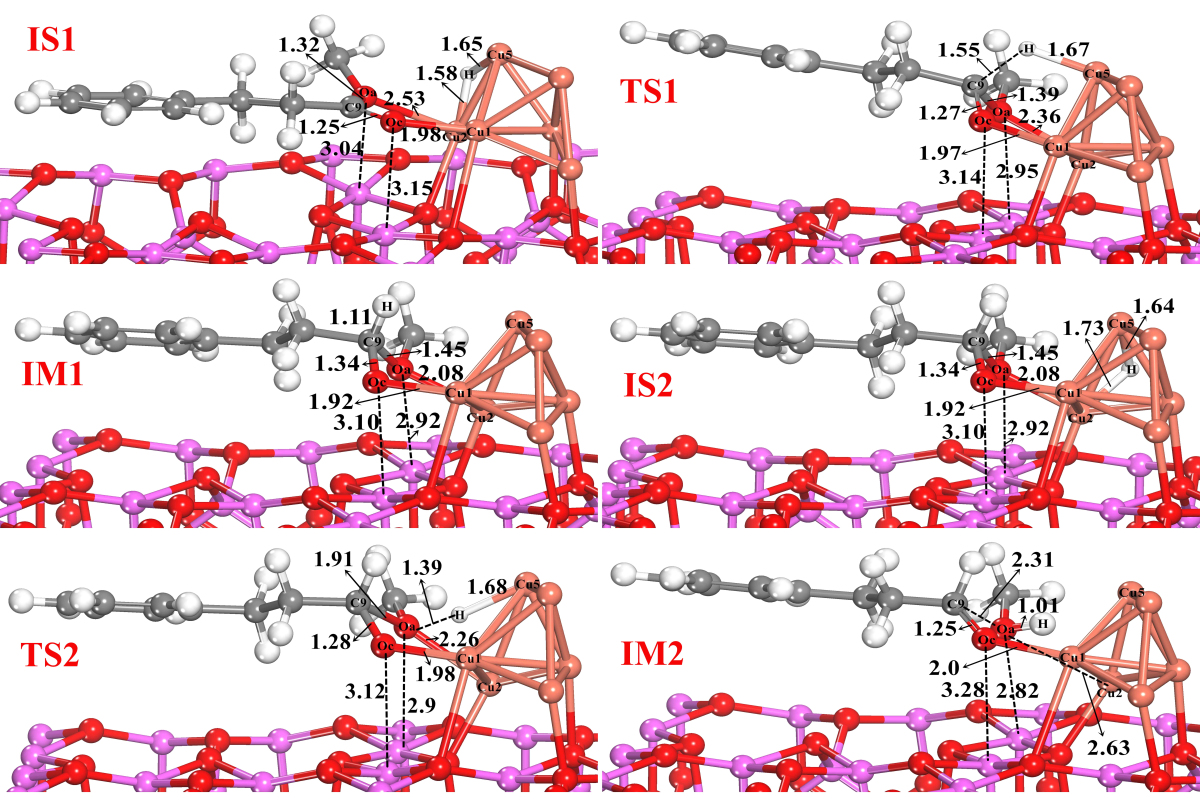


**Figure S6.** The structures for H_ad_ transferring from Cu to C=O on Cu_6_/γ-Al_2_O_3_(100) in step III, initial state of co-adsorbed 3-phenylpropionic acid methyl ester (3-p-a-me) and H (IS1), structure of transition state (TS1), structure of hemiacetal intermediate (IM1). The structures for the cleaving of C–O bond in hemiacetal by reaction with activated H on Cu_6_/γ-Al_2_O_3_(100) in step IV, initial state of co-adsorbed hemiacetal and H (IS2), structure of transition state (TS2), structure of intermediate (IM2). (Bond lengths are reported in Å).


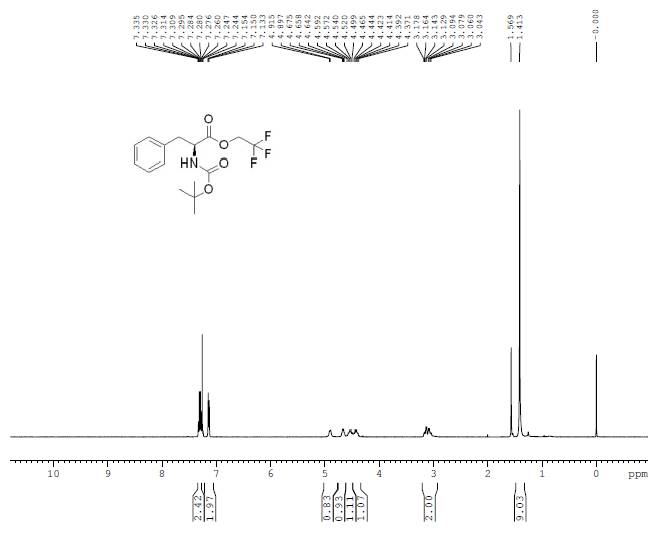


**Figure S7.** ^1^H NMR spectrum of N-Boc-L-Phenylalanine Trifluoroethyl Ester. ^1^H NMR(CDCl_3_): 7.13–7.34 (5H, m, Ph-H), 3.40–3.68 (2H, m, -CH_2_-O-), 3.16 (1H,m, -CH-N-), 2.55–2.84 (2H, m, CH_2_-Ph), 2.29 (2H, b, -NH_2_).


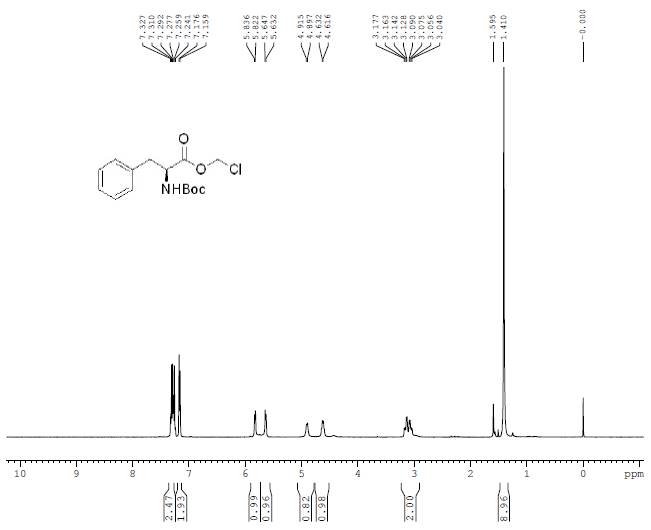


**Figure S8.** ^1^H NMR spectrum of N-Boc-L-Phenylalanine Chloromethyl Ester. ^1^H NMR (CDCl_3_): 7.16～7.33 (5H, m, Ph-H), 5.63～5.84 (2H, m, -CH_2_-O-), 4.62～4.63 (1H, d, -CH-N-), 3.04～3.18 (2H, m, CH_2_-Ph) , 4.90～4.92 (1H, d, -NH), 1.41 (9H, s, O-(CH_3_)_3_).


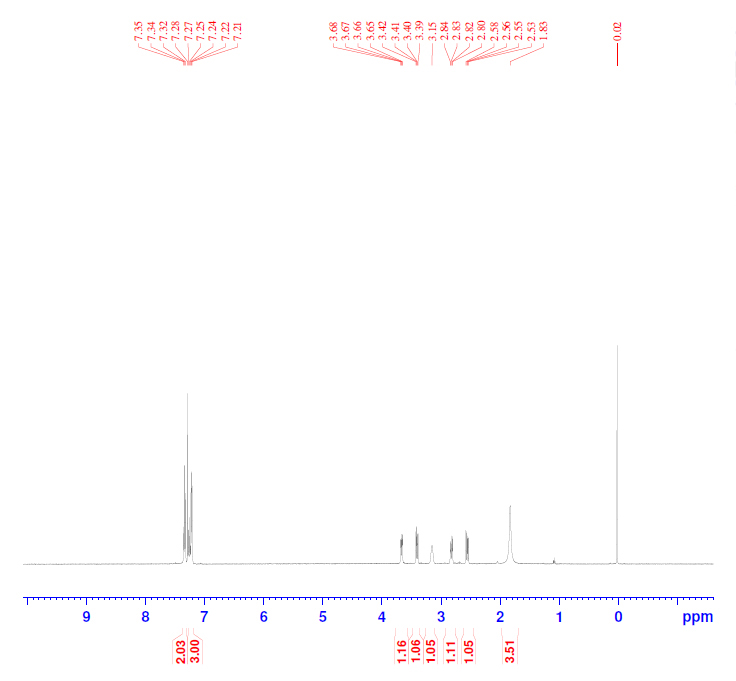


**Figure S9.** ^1^H NMR spectrum of product L-phenylalaninol. ^1^H NMR (CDCl_3_): 7.21～7.35 (5H, m, Ph-H), 3.42～3.68 (2H, m, -CH_2_-O-), 3.15 (1H, s, -CH-N-), 2.53～2.84 (2H, m, CH_2_-Ph) , 1.83 (2H, b, -NH_2_).


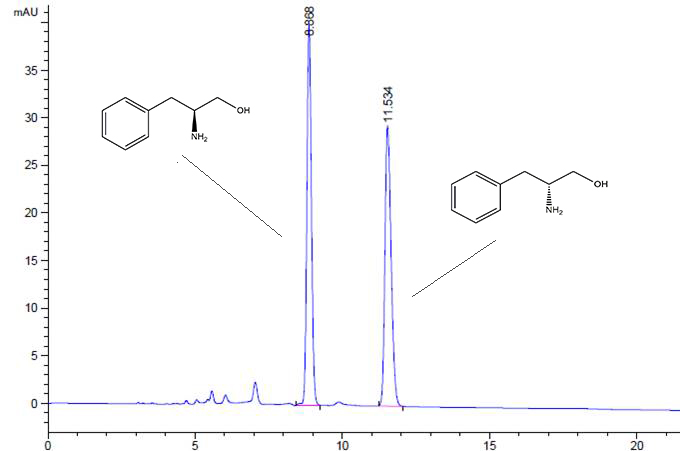


**Figure S10**. The chiral HPLC chromatogram of L-phenylalaninol and D-phenylalaninol mixture. The resolution of the enantiomers was 13.68, which indicates that the enantiomers of phenylalaninol were successfully resolved under the conditions used.

**
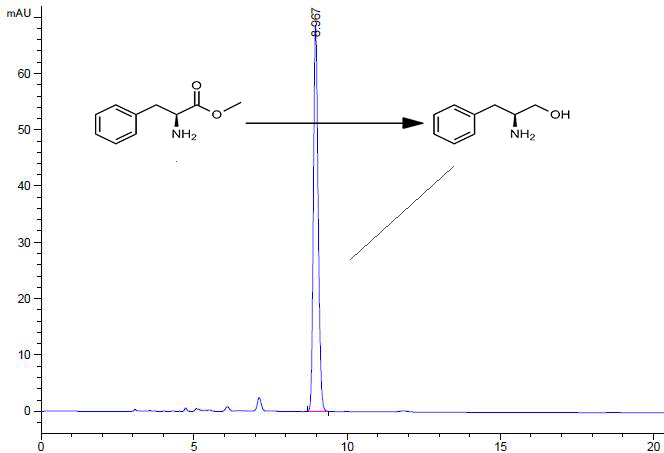
**

**Figure S11**. HPLC chromatogram of phenylalaninol product synthesized by hydrogenation of L-Phenylalaninate methyl ester.

**
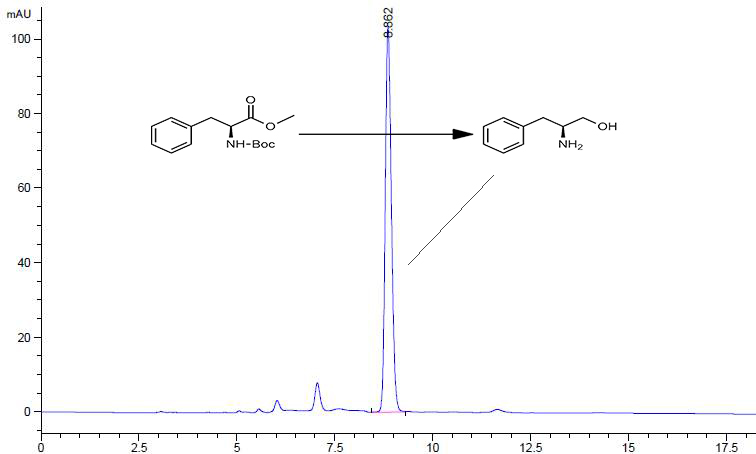
**

**Figure S12**. HPLC chromatogram of phenylalaninol product synthesized by hydrogenation of N-Boc-L-Phenylalaninate methyl ester.

**
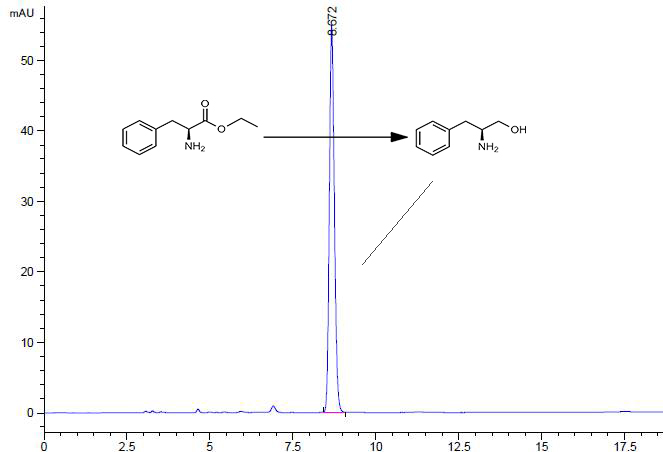
**

**Figure S13**. HPLC chromatogram of phenylalaninol product synthesized by hydrogenation of L-Phenylalaninate ethyl ester.

**
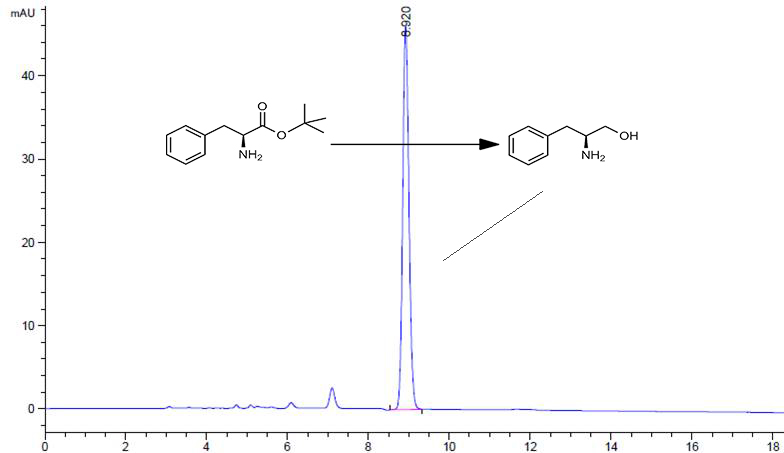
**

**Figure S14.** HPLC chromatogram of phenylalaninol product synthesized by hydrogenation of L-Phenylalaninate t-butyl ester.

**
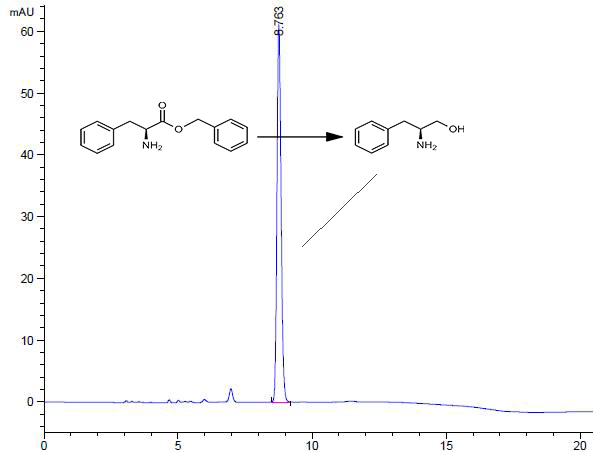
**

**Figure S15**. HPLC chromatogram of phenylalaninol product synthetized by hydrogenation of L-Phenylalaninate benzyl ester.

**
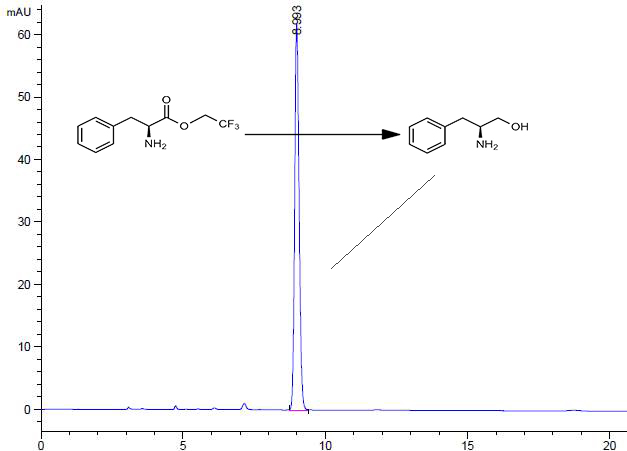
**

**Figure S16**. HPLC chromatogram of phenylalaninol product synthesized by hydrogenation of L-Phenylalaninate trifluoroethyl ester.

**
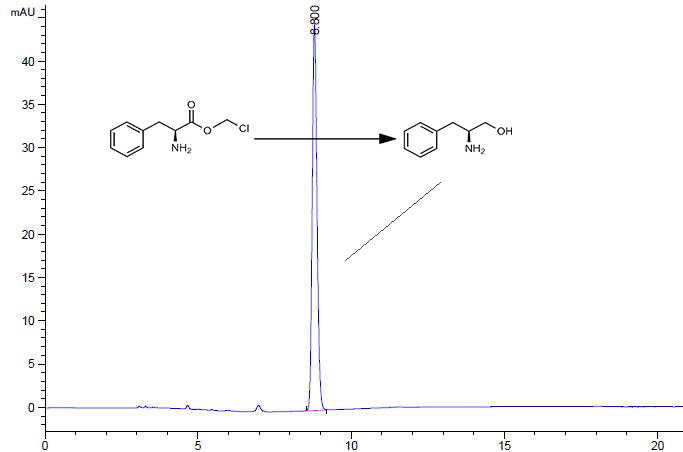
**

**Figure S17**. HPLC chromatogram of phenylalaninol product synthesized by hydrogenation of L-Phenylalaninate chloromethyl ester.


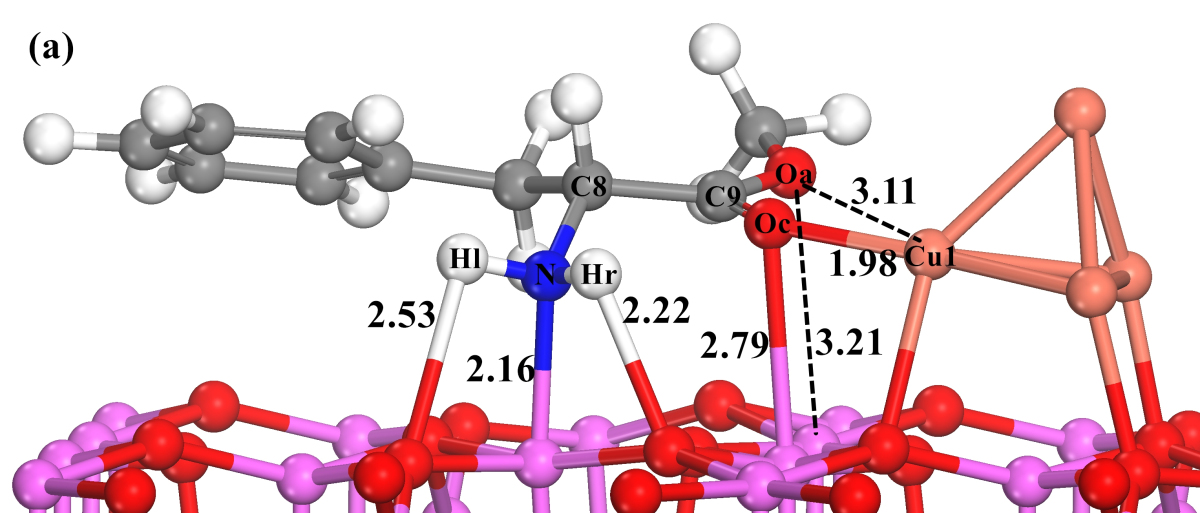


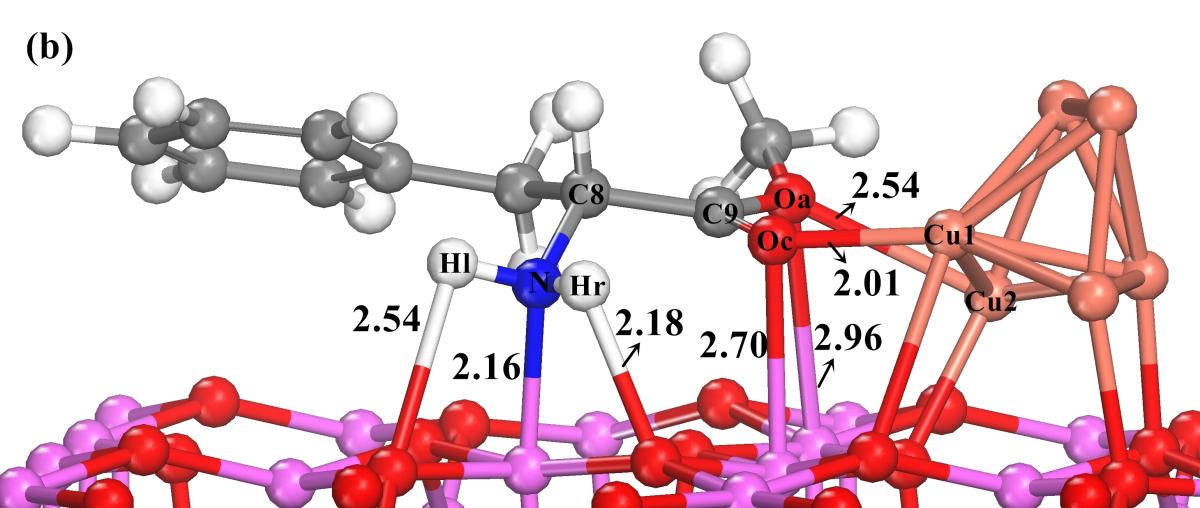


**Figure S18.** Optimized configuration of L-p-me adsorbed on (a) Cu_4_/γ-Al_2_O_3_(100) and (b) Cu_6_/γ-Al_2_O_3_(100). The adsorption energy of L-p-me adsorbed on Cu_4_/γ-Al_2_O_3_ (100) was -0.91 eV.
